# Reviews and Book Notices

**Published:** 1883-09

**Authors:** 


### Reviews and Book Notices.

ARTICLE XV.—ON THE RELATIONS OF MICRO-ORGANISMS TO DISEASE. The Cartwright Lectures, 1883. By WILLIAM T. BELFIELD, M.D. Wm. Wood & Co. W. T. Keener.

As a topic for the Cartwright lectures, intended as they were to enlighten a large part of the profession on some recent advances, no more fitting theme could have been chosen than the one selected by Dr. Belfield.

There is no one question in the whole department of medicine of greater importance to all physicians, no matter what their practice, of more decided revolutionary influence upon our beliefs and notions than the one: how far does the germ theory extend?

Moreover, there is perhaps no subject as thoroughly misunderstood in this country as the topic of these lectures.

The manner in which Dr. Belfield solved the task imposed upon himself is highly creditable. After an introduction to his subject, he describes bacteria in general, and then fully explains how to search for them and cultivate them, exposing at the same time the errors into which different observers have fallen.

He then discusses the relation of bacteria to surgical infections, and the theory of antiseptic surgery.

The third lecture deals fully with tuberculosis, and the fourth with anthrax, protective vaccination in that disease, and further interesting generalities. Finally, an appendix sums up the diseases in which bacteria are found as constant elements, while another explains the technique for detecting them.

While these lectures can hardly introduce a student fully into our present knowledge of bacteria, they serve admirably to place the topic in a lucid and comprehensive way before physicians partly acquainted with the details. When we reflect upon the amount of prejudice the germ theory encounters in this country, it seems proper, that the lecturer should have assumed a polemic, and even an aggressive tone. Although this style may not help to conciliate his opponents, it cannot be said that Dr. Belfield is unjust in his remarks. Sarcasm is one of the best weapons against ignorance, especially, when so well put as in these lectures.

A reviewer can of course find fault with any work. In this case it might be said, that some parts would have gained, especially the chapter on surgical infections, by a more systematic arrangement, with greater emphasis laid on the fact that the bacteria causing different diseases are different and distinct species.

Although this point is mentioned repeatedly, and indeed assumed throughout as an axiom, it is not insisted upon enough in the very places, where most confusion exists in the minds of the profession. For instance, it is yet urged against antiseptic surgery, even by a man like Lawson Tait, that bacteria are not known to cause putrefaction of the living tissues. This current misunderstanding shows, that even writers, who claim to be well informed, do not take into account sufficiently the difference in the action of different species of bacteria. Most pathogenic bacteria which can vegetate in the living tissues and injure them, do not cause putrefaction even in the dead soil, in which they may be cultivated. Again, the lecturer might have fortified his position better by referring more in detail to the recent positive demonstrations of the parasitic nature of erysipelas, gonorrhœa, trachoma, and suppurative inflammations in general.

Taking the book as a whole, however, it embodies so much of the recent research in this field, and presents it in such a fair ritical light and such an attractive style, that it cannot be too strongly recommended to that large part of the profession who have not yet given the subject the proper recognition. H. G.

---

ARTICLE XVI.—DISEASES OF THE EAR IN CHILDREN. BY VON TOPVALTSCH, Würzburg.

This volume of 165 pages forms a portion of Gerhardt's *Handbuch der Kinderkrankheiten;* it treats in a very clear and thorough manner of—

I. Diseases of the External Ear.

II. Diseases of the Middle Ear, Tympanum, Eustachian Tube, and Mastoid.

III. Foreign bodies in the Ear.

IV. Diseases of the Inner Ear or Labyrinth.

V. Deaf Mutism.

E. P. D.

---

AMERICAN DERMATOLOGICAL ASSOCIATION.

The Seventh Annual Meeting will be held at the Sagamore House, Green Island, Lake George, on Wednesday, Thursday, and Friday, August 29, 30, and 31. Papers will be read by the following gentlemen:

Dr. Piffard, Treatment of Acne; Dr. Hyde, A Study of the Coincidence of Syphilitic and Non-Syphilitic Affections of the Skin; Dr. Graham, General Exfoliative Dermatitus; Dr. Stelwagon, Impetigo Contagiosa; Dr. Robinson, Alopecia Areata; Dr. Duhring, 1. On the Value of a Lation of Sulphide of Zinc in the Treatment of Lupus Erythematosus. 2. Report of a Case of Ainhum with Microscopic Examination; Dr. Atkinson, A Case of Multiple Cachectic Ulceration; Dr. Sherwell, 1. Pseudo-Psoriasis of the Palm. 2. Malignant Papillary Dermatitis; Dr. Bulkley, 1. A Hitherto Undescribed Vegetable Parasite found on the Human Skin. 2. A Clinical and Experimental Study on Pruritus; Dr. Van Harlingen, Experiments in the Use of Napthol.

ARTHUR VAN HARLINGEN, M.D., Secretary.

DR. S. J. JONES has beeen out of town for a month, visiting Saratoga and the White Mountains in the East. He was in attendance upon the Ophthalmological Society.

PUBLICATION office of the JOURNAL has been removed to northwest corner State and Madison. The library of the Association is still at 188 South Clark street.

DR. J. N. HYDE will return to the city about September 1.

